# Influence of dose calculation algorithms on the helical diode array using volumetric‐modulated arc therapy for small targets

**DOI:** 10.1002/acm2.14307

**Published:** 2024-02-16

**Authors:** Tomohiro Ono, Hideaki Hirashima, Takanori Adachi, Hiraku Iramina, Takahiro Fujimoto, Megumi Uto, Mitsuhiro Nakamura, Takashi Mizowaki

**Affiliations:** ^1^ Department of Radiation Oncology and Image‐Applied Therapy Kyoto University Graduate School of Medicine Kyoto Japan; ^2^ Division of Clinical Radiology Service Kyoto University Hospital Kyoto Japan; ^3^ Department of Advanced Medical Physics Graduate School of Medicine Kyoto University Kyoto Japan

**Keywords:** Acuros XB, coarser dose resolution, GPR, small‐field irradiation, VMAT

## Abstract

**Background:**

For patient‐specific quality assurance (PSQA) for small targets, the dose resolution can change depending on the characteristics of the dose calculation algorithms.

**Purpose:**

This study aimed to evaluate the influence of the dose calculation algorithms Acuros XB (AXB), anisotropic analytical algorithm (AAA), photon Monte Carlo (pMC), and collapsed cone (CC) on a helical diode array using volumetric‐modulated arc therapy (VMAT) for small targets.

**Materials and methods:**

ArcCHECK detectors were inserted with a physical depth of 2.9 cm from the surface. To evaluate the influence of the dose calculation algorithms for small targets, rectangular fields of 2×100, 5×100, 10×100, 20×100, 50×100, and 100×100 mm^2^ were irradiated and measured using ArcCHECK with TrueBeam STx. A total of 20 VMAT plans for small targets, including the clinical sites of 19 brain metastases and one spine, were also evaluated. The gamma passing rates (GPRs) were evaluated for the rectangular fields and the 20 VMAT plans using AXB, AAA, pMC, and CC.

**Results:**

For rectangular fields of 2×100 and 5×100 mm^2^, the GPR at 3%/2 mm of AXB was < 50% because AXB resulted in a coarser dose resolution with narrow beams. For field sizes > 10×100 mm2, the GPR at 3%/2 mm was > 88.1% and comparable for all dose calculation algorithms. For the 20 VMAT plans, the GPRs at 3%/2 mm were 79.1 ± 15.7%, 93.2 ± 5.8%, 94.9 ± 4.1%, and 94.5 ± 4.1% for AXB, AAA, pMC, and CC, respectively.

**Conclusion:**

The behavior of the dose distribution on the helical diode array differed depending on the dose calculation algorithm for small targets. Measurements using ArcCHECK for VMAT with small targets can have lower GPRs owing to the coarse dose resolution of AXB around the detector area.

## 1 INTRODUCTION

Intensity‐modulated radiotherapy (IMRT) and volumetric‐modulated arc therapy (VMAT) achieve more precise and efficient delivery to tumors and have been used in several facilities. Patient‐specific quality assurance (PSQA) plays an important role in ensuring the safety of VMAT treatment.^1^, ^2^ In a general measurement‐based PSQA procedure, a treatment plan is computed using a homogeneous phantom to calculate the dose in the measurement tool geometry, and the dose is then measured using the measurement tool phantom. The treatment plan quality is determined based on the agreement between the calculated doses and the doses measured using the gamma passing rate (GPR). The common reason for the lower GPR is the uncertainty between the calculated and measured doses. However, the lower GPR values could be attributed to detector characteristics and dose calculation algorithms.^3^, ^4^

As a measurement tool for PSQA, the American Association of Physicists in Medicine (AAPM) Task Group 218 (AAPM‐TG218) has summarized vendor questionnaires on IMRT and VMAT PSQAs.^1^ In particular, ArcCHECK (Sun Nuclear Corporation, Melbourne, Florida, USA) and Delta4 (ScandiDos, Uppsala, Sweden) are designed to measure VMAT beams over the entire area. Their measurement tools have multidiode array detectors and are widely used as efficient tools for PSQA. AAPM‐TG 218 also recommends tolerance and action limits using GPR, and several facilities have introduced relevant criteria.^5^ However, in the case of stereotactic radiotherapy (SRT) for small targets, the actual dose may not be properly evaluated due to detector characteristics.^6^, ^7^, ^8^ Recently, the AAPM‐TG 219 introduced an independent calculation‐based method for IMRT and VMAT.^2^ This calculation‐based PSQA method assesses the dose variation between two different dose calculation algorithms. Calculation‐based PSQA is increasingly used in adaptive radiotherapy. Thus, it is necessary to understand that PSQA also depends on dose calculation algorithms.

For measurement‐based PSQA, although dose calculations are performed with a homogeneous phantom, the dose resolution of the dose calculation algorithm has a significant effect on the GPR for small targets.^9^ Therefore, the grid size should be finer in the treatment planning of small targets.^10^, ^11^ However, apart from the specified dose grid size, the dose resolution has different characteristics for each dose calculation algorithm. An anisotropic analytical algorithm (AAA, Varian Medical Systems, Palo Alto, California, USA) and a collapsed cone (CC) can cause a discretized dose distribution depending on the control point, and this effect is more noticeable for narrow beams.^12^ Even in more precise dose calculation algorithms, the relative uncertainty would be higher in the low‐dose region with Monte Carlo (MC),^13^ and a coarser dose resolution outside the primary volume of interest (PVOI) would be observed with Acuros XB (AXB, Varian Medical Systems).^14^ For ArcCHECK in particular, because the detectors are located farther from the center, their influence on the GPR is significant when using VMAT for small targets.

This study aimed to evaluate the influence of the dose distribution calculated by four types of dose calculation algorithms, namely, AXB, AAA, MC, and CC, on ArcCHECK, when using small‐field irradiation. Dose distributions were evaluated using rectangular multileaf collimator (MLC) fields and clinical VMAT plans with small targets, and their effects on the GPR were investigated.

## 2 METHODS

### 2.1 Measurement tool: ArcCHECK

ArcCHECK is a helical polymethylmethacrylate phantom with a three‐dimensional (3D) array of 1386 diode detectors with a 1 cm spacing.^15^ It has an outer diameter of 26.6 cm, and its detectors are inserted at a physical depth of 2.9 cm from the surface. The ArcCHECK measured dose is obtained from near the surface, and not from the center of the phantom. The dose distribution was analyzed using SNC Patient Software (version 6.6; Sun Nuclear). According to the SNC Patient Reference guide, the ArcCHECK phantom mass density was set to 1.18 g/cm^3^ for AXB and MC, and its relative density was set to 1.15 g/cm^3^.^15^ For AXB, the dose reporting mode was dose‐to‐water in medium according to the reporting mode evaluation described by Hirashima et al.^16^ For pMC, the dose reporting mode was dose‐to‐medium in medium because RayStation does not allow the dose reporting mode of dose‐to‐water in medium.

### 2.2 Dose calculation algorithm

Measurement‐based PSQA was performed using four types of dose calculation algorithms: AXB, AAA, pMC, and CC. AXB and AAA were supported by Eclipse (version 16.1.2; Varian Medical Systems). In contrast, pMC and CC were supported by RayStation (version 10A SP1; RaySearch Medical Laboratories AB, Stockholm, Sweden). The dose calculation algorithms are categorized into three types, namely, types “a,” “b,” and “”c^17^, ^18^, ^19^, ^20^ Type “a” does not account for the lateral transport of secondary electrons and is considered less accurate. Type “b” provides more accuracy in low‐density heterogeneous regions, taking convolution accuracy into consideration. Type “c” offers greater accuracy than types “a” and “c,” especially in regions around heterogeneity. In this study, AAA and CC were categorized as type “b,” while AXB and pMC were categorized as type “c.” For all dose calculations, the dose grid size was set to 1×1×1 mm^3^. The details of each dose calculation algorithm are presented in the following sections.

#### 2.2.1 Acuros XB (AXB)

**FIGURE 1:**
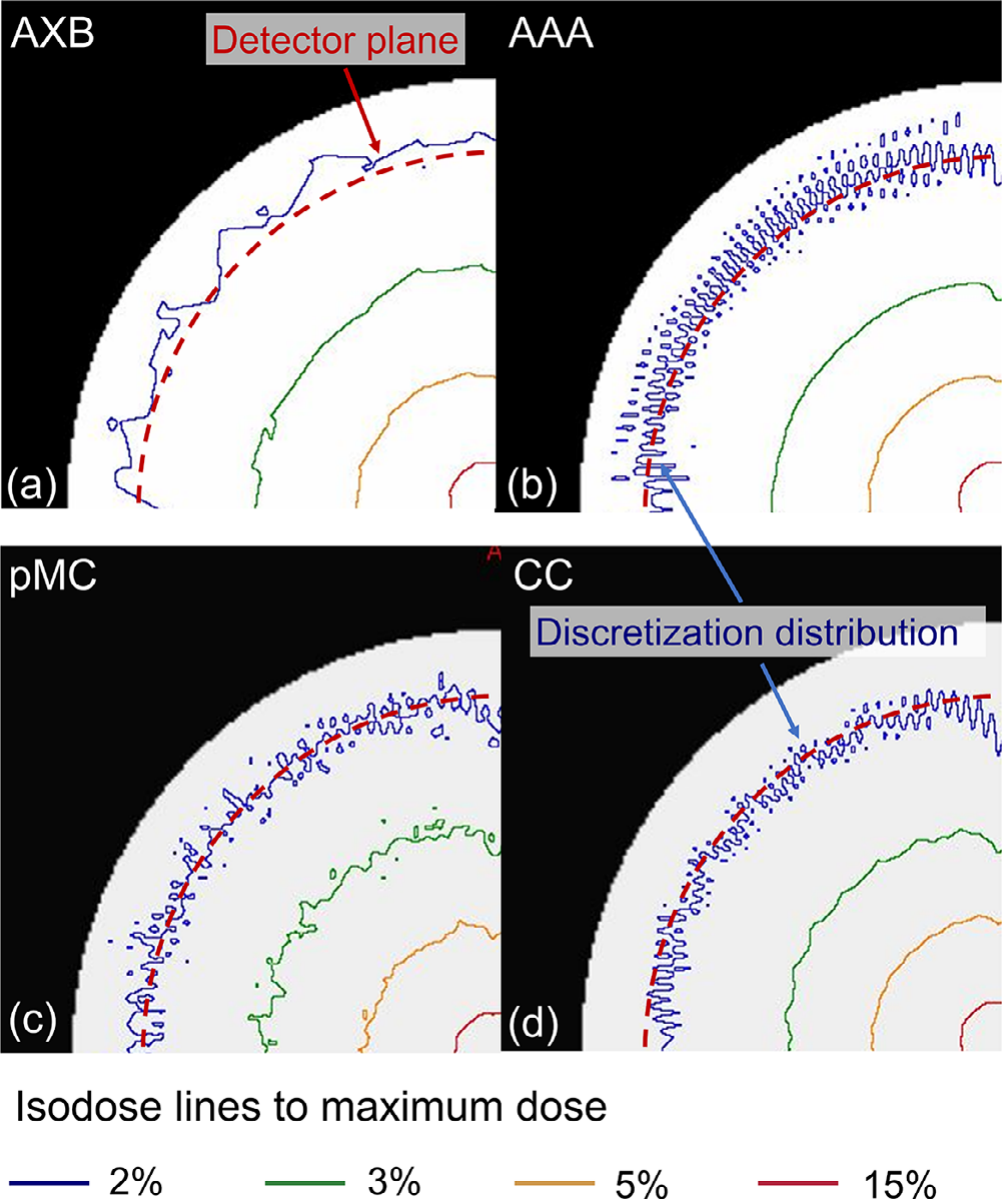
Example of dose distributions on the ArcCHECK phantom with a field size of 2×100 mm^2^ for (a) AXB, (b) AAA, (c) pMC, and (d) CC. Isodose lines, which include 2%, 3%, 5%, and 15% to maximum dose are shown. Red dotted circles show the detector plane of ArcCHECK. AXB, Acuros XB; AAA, anisotropic analytical algorithm; pMC, photon Monte Carlo; CC, collapsed cone.

AXB is a grid‐based linear Boltzmann transport equation solver that accounts for tissue heterogeneity.^19^ For calculation efficiency, spatial discretization is adapted to achieve a higher spatial resolution inside the beam, with reduced resolution in the lower‐dose and lower‐gradient regions outside the beam penumbra. AXB employs a finer resolution to calculate the electron fluence inside the PVOI and a coarser dose resolution outside the PVOI.^14^ The coarser dose resolution is twice the user‐defined dose resolution. The PVOI is defined as the volume at which the dose is estimated to be ≥15% of the maximum dose for plans containing arc fields. Figure 1a shows an example of isodose lines on the ArcCHECK phantom with a field size of 2×100 mm^2^ for AXB. Isodose lines, which include 2%, 3%, 5%, and 15% to maximum dose are shown. Here, the coarser dose resolution was observed at the 15% isodose line, and the 2% isodose line was observed around the detector plane. It can also be observed that the resolution was coarser at lower doses.

#### 2.2.2 Anisotropic analytical algorithm (AAA)

AAA is a 3D pencil beam convolution/superposition algorithm that uses separate MC‐derived models for primary photons, scattered extrafocal photons, and electrons scattered from beam‐limiting devices.^21^, ^22^ It accounts for tissue heterogeneity anisotropically in the entire 3D neighborhood of an interaction site using photon scatter kernels in multiple lateral directions with beamlets. The depth coordinates are measured from the intersection point of the central fanline and the skin in the beamlet coordinate system. Thus, in the calculation region near the surface, the deposited dose is affected by the beamlet coordinate system rather than the patient coordinate system and this causes a discretization distribution, as shown in Figure 1b. Here, discretization distributions caused by the deposited dose were observed at the 2% isodose line.

#### 2.2.3 Photon Monte Carlo (pMC)

The pMC is a statistical model that generates particles at defined locations and follows them; it is reported to be a class II condensed history implementation with the same dual virtual source head model that is used in RayStation's CC dose calculation algorithm.^23^ Transport in a patient's geometry is inspired by the EGSnrc. By simulating a sufficiently large number of particles, pMC can predict the dose deposition outcomes, including the near‐surface region, within the media for a preset uncertainty. In this study, the uncertainty was set to 1% for the evaluation of rectangular fields, and to 2% for the clinical VMAT plans. The number of particles depends on the field size. In addition, uncertainty is in general larger in the build‐up region, as fewer histories contribute to dose deposition in the region, as shown in Figure 1c.

#### 2.2.4 Collapsed cone (CC)

CC algorithms use the superposition–convolution method based on the basic principle to separately consider the primary photon transport, as well as the secondary transport of photons and electrons that are created in the primary photon interaction.^24^ Electron contamination is computed using a pencil beam algorithm and is added to the photon dose considering tissue heterogeneity. CC is implemented in various commercial treatment planning systems as a more accurate dose calculation algorithm compared to simpler type “a” algorithms. In CC, the deposited dose is affected by the beamlet coordinate system, similar to AAA, and causes a discretized distribution near the phantom surface, as shown in Figure 1d.

### 2.3 Evaluation of calculated and measured doses with rectangular fields

To evaluate the effect of dose distribution near the phantom surface, small rectangular fields were irradiated and measured using ArcCHECK. Rectangular MLC fields, including 2×100, 5×100, 10×100, 20×100, 50×100, and 100×100 mm^2^, were created using the HD120 MLC (leaf width: center, 2.5 mm; peripheral, 5 mm) of TrueBeamSTx (Varian Medical Systems). Each plan was created as one full arc with a static MLC field. Dose calculations were performed using AXB, AAA, pMC, and CC with 10 MV flattening‐filter‐free (FFF) energy and 500 monitor units (MUs). The GPRs at 5%/1 mm, 3%/2 mm, and 3%/3 mm were evaluated for the four dose calculation algorithms. The GPRs were evaluated for areas receiving isodoses > 10% using the global difference approach.

### 2.4 Evaluation of clinical volumetric‐modulated arc therapy (VMAT) plans

Twenty VMAT plans designed as SRT techniques between April 2017 and January 2023 at a single institution were retrospectively selected. The VMAT plans with < 85% GPR (3%/3 mm) were arbitrarily selected for this study. The clinical sites included the brain (n = 19) and spine (n = 1), and cases of brain metastases included multiple brain metastases. The mean and standard deviation of the planning target volumes (PTVs) was 1.36 ± 1.75 cc (maximum, 6.78 cc; minimum, 0.12 cc), and that of the equivalent diameter was 11.7 ± 5.2 mm (maximum, 23.0 mm; minimum, 0.7 mm). All VMAT plans were created with a combination of coplanar and non‐coplanar arcs using Eclipse, and were calculated using AXB with 10 MV‐FFF energy. Subsequently, the VMAT plans, which had the same MLC positions and MUs for each control point, were recalculated using the AAA, pMC, and CC. To evaluate the dosimetric accuracy in more detail, PSQA was performed for per‐arc measurements. Thus, the actual number of VMAT beams was 61. Measurement‐based PSQA was performed using ArcCHECK with the acrylic plug. To evaluate the quality of the VMAT plans, the modulated complexity score for VMAT (MCS_v_) and the average aperture area (AA) were evaluated.^25^, ^26^ Dose calculations were performed using AXB, AAA, pMC, and CC. The GPRs at 5%/1 mm, 3%/2 mm, and 3%/3 mm were evaluated for the four dose calculation algorithms.

## 3 RESULTS

### 3.1 Evaluation of rectangular fields

**FIGURE 2:**
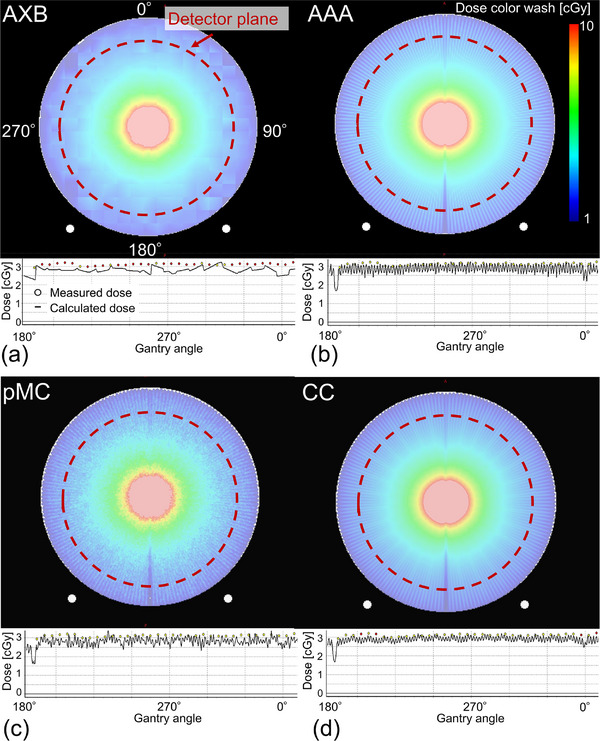
Dose distributions and dose profiles with a field size of 2×100 mm^2^ for (a) AXB, (b) AAA, (c) pMC, and (d) CC. Red dotted circles show the detector plane of ArcCHECK. AXB, Acuros XB; AAA, anisotropic analytical algorithm; pMC, photon Monte Carlo; CC, collapsed cone.

Figure 2 shows examples of dose distributions and dose profiles with a field size of 2×100 mm^2^ for AXB, AAA, pMC, and CC. The dotted circles indicate the detector placement at 2.9 cm from the surface of ArcCHECK. The color bar range was set equal to the analysis range of the GPR in the detector plane. For AAA, pMC, and CC, the discretized dose distribution, depending on the control points, was observed around the detector plane area. In contrast, a coarser dose resolution was observed with AXB around the detector plane area. Table 1 shows the GPRs of the rectangular fields at 5%/1 mm, 3%/2 mm, and 3%/3 mm for AXB, AAA, pMC, and CC. The number of particles in pMC was 1.9×10^8^ to 1.6×10^9^. Particularly for narrow beams of 2×100 and 5×100 mm^2^, the GPRs of AXB were < 50% of those of the other dose calculation algorithms; however, for field sizes > 10×100 mm^2^, the GPRs were comparable for all dose calculation algorithms. Thus, the differences in GPR were attributed to variations in surface area characteristics resulting from each dose calculation algorithm and smaller field sizes, with 5×100 mm^2^ identified as the relevant threshold of smaller field sizes.

**TABLE 1:** GPRs of rectangular fields for AXB, AAA, pMC, and CC.

	GPR (5%/1 mm) [%]				GPR (3%/2 mm) [%]				GPR (3%/3 mm) [%]			
Field size [mm^2^]	AXB	AAA	pMC	CC	AXB	AAA	pMC	CC	AXB	AAA	pMC	CC
2 × 100	27.5	78.7	61.9	66.2	30.1	97.7	90.0	79.9	40.1	99.9	98.7	91.0
5 × 100	46.5	92.7	78.9	77.2	41.8	97.0	84.7	83.4	54.8	98.8	96.5	95.9
10 × 100	82.3	97.9	62.4	98.0	89.1	97.6	88.1	97.8	95.8	98.4	99.0	98.7
20 × 100	84.4	99.1	74.8	95.9	89.4	97.9	90.3	89.0	97.0	98.3	99.6	97.6
50 × 100	99.5	99.9	78.6	97.4	98.7	98.8	87.7	99.6	99.5	99.7	99.0	99.9
100 × 100	99.6	100.0	91.3	98.2	99.9	99.9	90.5	99.9	99.9	100.0	99.5	100.0

Abbreviations: AAA, anisotropic analytical algorithm;AXB, Acuros XB; CC, collapsed cone; GPR, gamma passing rate.; pMC, photon Monte Carlo.

### 3.2 Evaluation of gamma passing rates (GPRs) for VMAT plans

**FIGURE 3:**
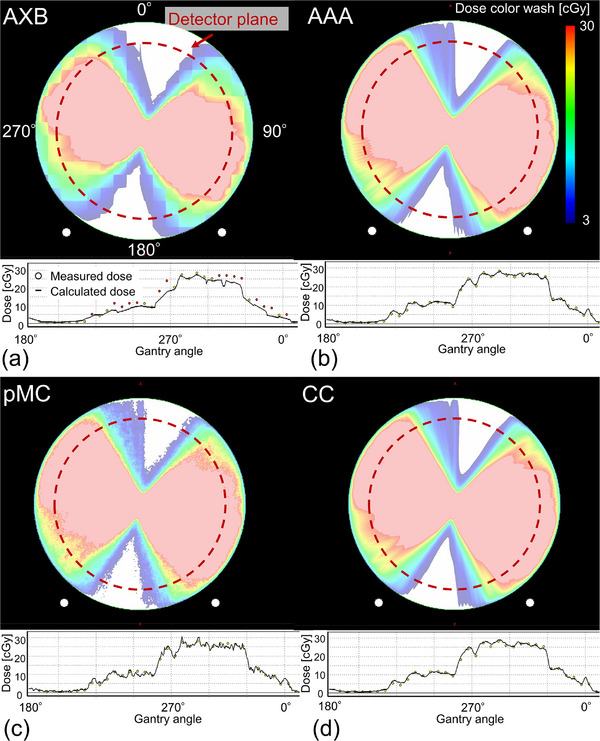
An example of dose distributions and dose profiles of the VMAT plan for (a) AXB, (b) AAA, (c) pMC, and (d) CC. Red dotted circles show the detector plane of ArcCHECK. AXB, Acuros XB; AAA, anisotropic analytical algorithm; pMC, photon Monte Carlo; CC, collapsed cone; VMAT, volumetric‐modulated arc therapy.

**FIGURE 4:**
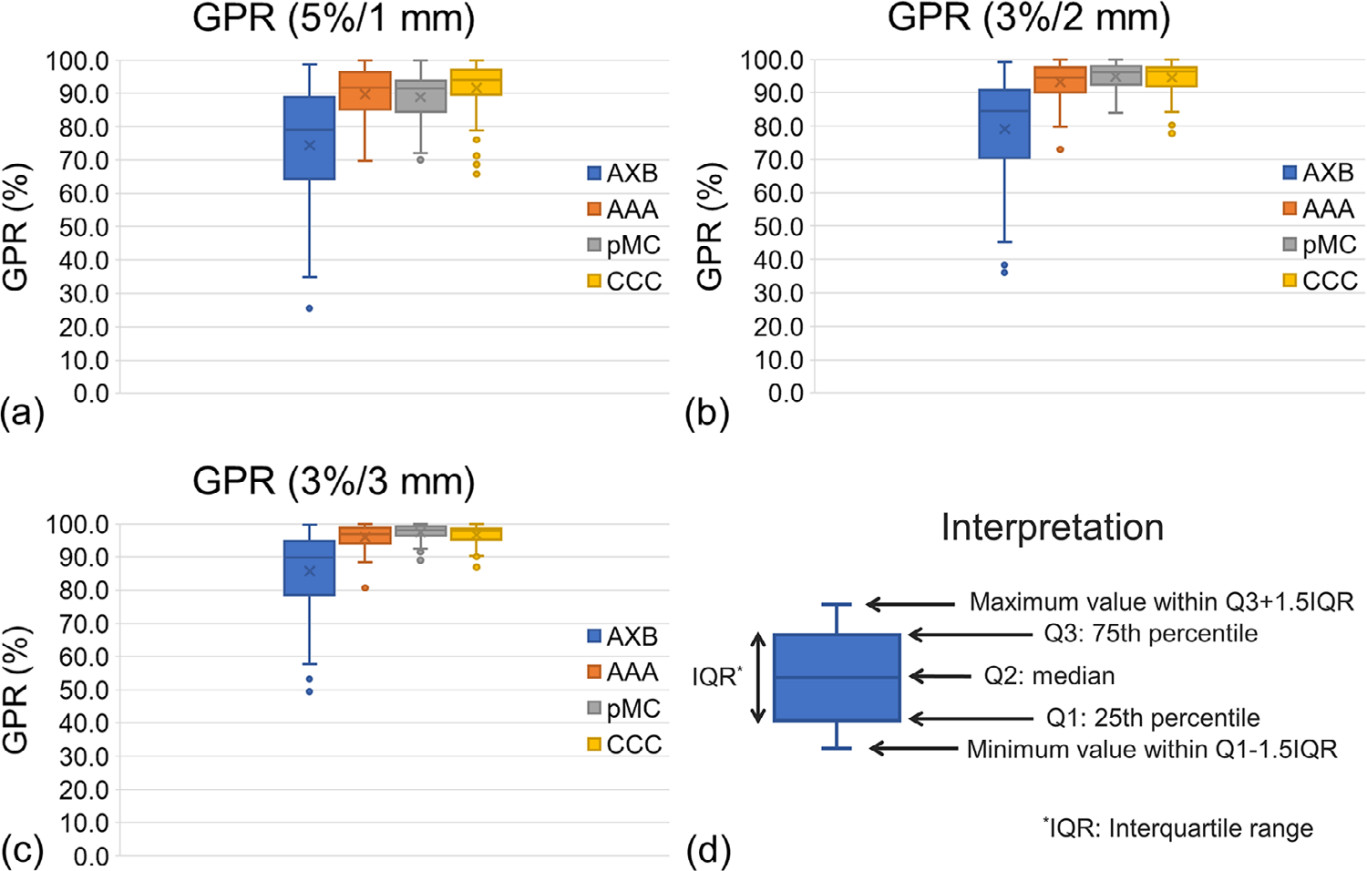
Boxplot of GPRs at (a) 5%/1 mm, (b) 3%/2 mm, and (c) 3%/3 mm for AXB, AAA, pMC, and CC. (d) Explanation of interpretation of boxplot. AXB, Acuros XB; AAA, anisotropic analytical algorithm; pMC, photon Monte Carlo; CC, collapsed cone; GPR, gamma passing rate.

Regarding the plan complexity parameters of 61 arcs, the MCS_v_ and AA were 0.14 ± 0.07 (range, 0.03−0.30) and 305.1 ± 249.7 (range, 66.6−1288.7) mm^2^, respectively. The maximum MCS_v_ (0.30) was observed with a single target volume (3.96 cc), whereas the minimum MCS_v_ (0.02) was observed with four PTVs of 0.15−0.35 cc. The maximum AA of 1288.7 cc was observed for 11 target plans, and the minimum AA of 66.7 mm^2^ was observed for a single target with a PTV of 0.30 cc. Figure 3 shows an example of the dose distributions of the VMAT plan on ArcCHECK geometry, as well as the comparison of dose profiles between the measured and calculated doses for AXB, AAA, pMC, and CC. Notably, the dose resolution was coarse around the surface of the ArcCHECK in AXB. In the AXB dose profile, the calculated dose was lower and coarser than the measured dose. For pMC, although scattered doses were observed around the detector plane, the profiles of the measured and calculated doses were similar. For AAA and CC, there were similar dose distributions owing to dose discretization around the detector plane, and the profiles were also similar between the measured and calculated doses. Figure 4 shows the boxplots of the GPRs at 5%/1 mm, 3%/2 mm, and 3%/3 mm for AXB, AAA, pMC, and CC. The GPRs values were 74.4 ± 17.8%, 89.7 ± 8.2%, 88.9 ± 6.8%, and 91.6 ± 7.9% at 5%/1 mm; 79.1 ± 15.7%, 93.2 ± 5.8%, 94.9 ± 4.1%, and 94.5 ± 4.7% at 3%/2 mm; as well as 85.7 ± 12.5%, 95.9 ± 3.7%, 97.3 ± 2.6%, and 96.6 ± 3.2% at 3%/3 mm for AXB, AAA, pMC and CC, respectively. The GPRs of ABV were lower than those of the other calculation algorithms owing to the coarse dose resolution.

## 4 DISCUSSION

This study evaluated the influence of the calculated dose distribution on a helical diode array with a small field. The behavior of the dose distribution in the outer region of the irradiated field center differed depending on the dose calculation algorithm. Particularly in AXB, the dose resolution at the edges was coarser, which led to a worse GPR. In the case of a large irradiation field plan, the detector plane area of ArcCHECK would be within a finer dose resolution in the PVOI; thus, the calculation grid would not be coarse. In the evaluation of plans with small targets, a worse GPR would be influenced by the detector characteristics and output uncertainties and by the calculated dose grid size.

The dependence of the field size on ArcCHECK was evaluated. Chaswal et al.

evaluated narrow beam aperture arcs for ArcCHECK‐measured doses and compared them with the dose calculated using AAA.^12^ They found that the GPRs at 3%/3 mm were 75.0%, 87.9%, and 96.1% for narrow‐beam apertures of 20×100, 30×100, and 50×50 mm^2^, respectively. Generally, the calculation of arcs relies on the discretization of continuous arcs into several static beams. Feygelman et al. indicated that the calculated dose away from the isocenter was dependent on the control point spacing.^27^ The current study also observed a discretized dose distribution in the detector plane area of ArcCHECK using AAA and CC. Such discrete doses were not observed for AXB or pMC. However, AXB could cause a coarser dose resolution outside the PVOI when using small‐field irradiation.

The GPR is related to the plan's complexity.^25^, ^28^, ^29^, ^30^ In particular, the MCS_v_ tends to be lower for VMAT plans with small targets.^31^ The current study also observed a low MCS_v_ value of 0.14 ± 0.07 and a small AA of 305.1 ± 249.7 mm^2^ for the 20 VMAT plans. It is estimated that such a narrow beam would cause a discretized dose by AAA and CC, as well as a coarser dose resolution by AXB.

This study has a limitation that should be acknowledged: it did not provide a specific solution for the worsening resolution of AXB. As a method to mitigate the effects of a coarser dose resolution outside the PVOI, He et al. reported the usefulness of the short split arc method with AXB.^32^ It was found that the dose calculation by dividing one arc into several split arcs widened the PVOI region and prevented coarse dose resolution in the detector plane in ArcCHECK. However, considering the irradiation and optimization processes, treatment planning with a split arc is inefficient, and improvement of the AXB dose calculation algorithm is desirable. In addition, generally, PSQA should be performed using the dose calculation algorithm employed in the clinical plan. If the clinical plan is calculated using AXB, then PSQA should also use AXB. However, when performing PSQA for plans with small fields using AXB, coarse dose resolution may be observed. In such cases, we believe that conducting PSQA using other dose calculation algorithms instead of AXB is an available approach, provided that the other dose calculation algorithm and AXB are commissioned to have comparable dose accuracy.

## 5 CONCLUSION

The influence of dose calculation algorithms, including AXB, AAA, pMC, and CC, in small‐field irradiation was evaluated using the ArcCHECK detector. The behavior of the dose distribution on the helical diode array differed depending on different dose calculation algorithms for small targets. Measurements using ArcCHECK for VMAT with small targets can have a lower GPR owing to the coarse dose resolution of the AXB. Particularly, for ArcCHECK, where the detector is located farther from the irradiation center, the characteristics of the dose calculation algorithm have a significant effect on the GPR. In clinical practice, it should be noted that the GPR may not be accurately evaluated in PSQA performed with ArcCHECK for treatment plans with small fields due to the characteristics of the dose calculation algorithm rather than the characteristics of the detector.

## AUTHOR CONTRIBUTIONS

Tomohiro Ono, Hideaki Hirashima, Takanori Adachi, Hiraku Iramina, Takahiro Fujimoto, and Megumi Uto conceived of the presented idea and verified the analytical methods. Mitsuhiro Nakamura and Takashi Mizowaki helped supervise the project. All authors discussed the results and contributed to the final manuscript.

## CONFLICT OF INTEREST STATEMENT

The authors have no relevant conflicts of interest to disclose.

## ETHICS STATEMENT

This study (R1446) was approved by the Institutional Review Board of Kyoto University Hospital on January 30, 2018.
